# Book Reviews in Brief

**Published:** 1913-01-11

**Authors:** 


### Book Reviews in Brief.

HYGIENE. For Health Visitors, School Nurses, and Social Workers. By C. W. HUTT, M.A., B.C. (Cantab.). (London: P. S. King and Son. 1912. 7s. 6d. net.)

THIS book contains matter which may not be new to many, but is nevertheless arranged and classified so as to form an interesting and useful practical text-book for those interested in the social welfare and betterment of the poorer classes. Written by a medical officer of health, and dedicated to a health committee, it follows the examination syllabus of the Royal Sanitary Institute, and gives in a clear and lucid manner information on the various subjects required by health visitors.

PERFECT HEALTH FOR WOMEN AND CHILDREN. By ELIZABETH SLOAN CHESSER, M.B., Ch.B. (London: Methuen and Co., Ltd. 1912. Price 3s. 6d.)

THE desire of the laity for medical knowledge has always been a feature of modern civilisation, but until comparatively recently it must be admitted that this craving has been but indifferently catered for. At the present time, however, guides to health and guides in sickness may be fairly said to abound. A book on the preservation and regaining of health written by a lady doctor for women should have material advantages. In this volume by Dr. Chesser a very praiseworthy attempt has been made to impart a great deal of sound advice and many home truths to a class that badly needs instruction. It will be conceded that the women of to-day are steadily improving in the practice of elementary hygiene, and books of this nature find not only purchasers but students who heed. It is, therefore, of great importance that such guides should be written by those possessing first-hand knowledge and with a plentiful supply of common sense and the absence of any suggestion of fad. Dr. Chesser has succeeded in producing a volume which should prove exceedingly helpful. She preaches thoroughly the gospel that if we are ill it is probably our own fault and not a matter of luck. Much sensible advice about the health of women and children, not only regarding their bodies, but their minds also, is to be found in this volume. The second portion of the book deals with sick nursing in the home, and we are glad to note that medical advice is restricted to a safe limit, but yet serviceable and feasible. This emphasises the importance of having such books as these written by medical practitioners and not by hygienic experts, ex-matrons, or gymnastic professors.

DISEASES OF WOMEN. By T. G. STEVENS, M.D. (University of London Press: Henry Frowde and Hodder and Stoughton. 1912. Pp. 431. Price 15s net.)

THERE are many text-books upon this subject, and the best of those which have appeared in recent years are a good deal better than those which were the favourites a dozen years ago. When it is added that this one is, in our judgment, as good as the best that has hitherto been seen, it will be understood that it is excellent indeed. The arrangement is admirable, and the teaching is sound; the whole book betrays long experience of student needs, and is the best we know of to place in the hands of those starting the study of gynæcology. It is excellently produced, and a special word of praise is due to the splendid microphotographs, which really illustrate the text and are most valuable. Dr. Stevens is to be congratulated upon a work which has to compete for popularity against several first-rate rivals, but will certainly achieve a marked success. His text-book is good value for the price at which it is published.

TEXT-BOOK OF ANATOMY AND PHYSIOLOGY FOR NURSES. By ELIZABETH R. BUNDY, M.D. Second Edition, Revised and Enlarged. (London: J. and A. Churchill. 1912. Price 7s. 6d.)

THIS Text-Book is rather more ambitious than the average manual of anatomy intended for nurses; the illustrations are numerous, and a large number of them are well coloured, while, seeing that many of them are taken from a well-known treatise on anatomy, their accuracy and clearness are fully established. Without including too much detail, this book contains a fairly comprehensive description of the various structures composing the human body, and might, indeed, receive attention from medical students as an introduction to the standard text-books. In this edition some chapters have been added on physiology, making the book more completely serviceable to nurses. Although this Text-Book betrays its American origin, there is little to cavil at, and throughout use is made of the Basle anatomical nomenclature, which is slowly becoming the standard everywhere. The arrangement of the sections is excellent, the type beautifully clear and bold, and the text itself suitably explicit.

